# Laryngoscopy evaluation protocol for the differentiation of essential and dystonic voice tremor^☆^^☆☆^

**DOI:** 10.1016/j.bjorl.2015.11.001

**Published:** 2015-12-03

**Authors:** Bruno Teixeira de Moraes, Noemi Grigoletto de Biase

**Affiliations:** a Department of Otorhinolaryngology, Health Sciences, Universidade Federal de São Paulo (UNIFESP), São Paulo, SP, Brazil; b Department of Otorhinolaryngology, Universidade Federal de Pernambuco (UFPE), Recife, PE, Brazil

**Keywords:** Tremor, Essential tremor, Dystonia, Laryngoscopy, Tremor, Tremor essencial, Distonia, Laringoscopia

## Abstract

**Introduction:**

Although syndromes that cause voice tremor have singular characteristics, the differential diagnosis of these diseases is a challenge because of the overlap of the existing signs and symptoms.

**Objective:**

To develop a task-specific protocol to assess voice tremor by means of nasofibrolaryngoscopy and to identify those tasks that can distinguish between essential and dystonic tremor syndromes.

**Methods:**

Cross-sectional study. The transnasal fiberoptic laryngoscopy protocol, which consisted of the assessment of palate, pharynx and larynx tremor during the performance of several vocal and non-vocal tasks with distinct phenomenological characteristics, was applied to 19 patients with voice tremor. Patients were diagnosed with essential or dystonic tremor according to the phenomenological characterization of each group. Once they were classified, the tasks associated with the presence of tremor in each syndrome were identified.

**Results:**

The tasks that significantly contributed to the differential diagnosis between essential and dystonic tremor were /s/ production, continuous whistling and reduction of tremor in falsetto. These tasks were phenomenologically different with respect to the presence of tremor in the two syndromes.

**Conclusion:**

The protocol of specific tasks by means of transnasal fiberoptic laryngoscopy is a viable method to differentiate between essential and dystonic voice tremor syndromes through the following tasks: /s/ production, continuous whistling and reduction of tremor in falsetto.

## Resumo

**Introdução:**

Apesar de haver características próprias entre as síndromes causadoras do tremor vocal, o diagnóstico diferencial destas doenças é um desafio pela sobreposição de sinais e sintomas presentes.

**Objetivo:**

Desenvolver protocolo de tarefas específicas na avaliação do tremor vocal por nasofibrolaringoscopia e identificar aquelas que diferenciem as síndromes de tremor essencial e distônico.

**Método:**

Estudo transversal. O protocolo de nasofibrolaringoscopia, que consistiu na avaliação do tremor em palato, faringe e laringe durante execução de diversas tarefas fonatórias e não-fonatórias com características fenomenológicas distintas, foi aplicado em 19 pacientes com tremor vocal. Os pacientes foram diagnosticados como tremor essencial ou distônico a partir da caracterização fenomenológica de cada grupo. Uma vez classificados, determinou-se quais tarefas estavam associadas à presença de tremor nas diferentes síndromes.

**Resultados:**

As tarefas que contribuíram significativamente na distinção do tremor essencial e distônico foram a emissão /s/, assobio contínuo e redução do tremor no agudo, pois apresentaram-se fenomenologicamente diferentes quanto à presença do tremor entre as duas síndromes.

**Conclusão:**

O protocolo de tarefas específicas por nasofibrolaringoscopia é um método viável para diferenciar as síndromes de tremor vocal essencial e distônico por meio das tarefas: emissão /s/, assobio contínuo e redução do tremor no agudo.

## Introduction

Voice tremor can be present in defined syndromes when it is associated with other neurological signs (especially for limbs and head), or it is frequently described as an isolated voice tremor when the voice tremor is the only manifestation.^1^ Considering the phenomenology, the tremor can be present at rest or with action depending on the conditions present when the tremor occurs and the main clinical data that contribute to the syndromic diagnosis. This description also applies to the phonatory apparatus and can be evaluated by means of nasofibrolaryngoscopy, which allows a functional assessment during the performance of several tasks.^2^

Among the neurological syndromes with possible voice tremor manifestations, essential tremor is most prevalent followed by Parkinsonian tremor and dystonic tremor.^3^, ^4^ Despite being a relatively common entity, some case series show that Parkinson's disease rarely exhibits a laryngeal tremor.^5^, ^6^ Considering that tremor at rest is physiologically and clinically different from the others, the greatest diagnostic difficulty lies in distinguishing between essential and dystonic tremor.^7^

Essential tremor presents with vocal disorders in 11–30% of cases.^5^, ^8^, ^9^ The rhythmic oscillation of the palate, pharynx or larynx may be present during all tasks, including quiet breathing and speech, because these are the activities that require the maintenance of a definite laryngeal posture^10^; however, the possibility of relaxing the laryngeal muscles during quiet breathing is questionable.^11^

Koufman and Blalock (2004)^12^ proposed a classification of laryngeal dystonia, in which they describe dystonic tremor as a variation of the adductor laryngeal dystonia, wherein glottal hyper-adductions are rhythmic. It differs from the essential tremor, as it is typically more focal (usually without the involvement of other body structures) and task-specific, since it depends on phonatory activity for its onset, and is absent during quiet breathing.

Although there are specific characteristics among the syndromes that cause voice tremor, there is no sufficiently specific diagnostic method by which to differentiate these diseases because of their overlap of signs and symptoms.^5^ The existence of a protocol that considers the different situations in the evaluation by nasofibrolaryngoscopy and that allows the assessment of the phonatory apparatus as close as possible to the physiological state, would help in the analysis of the tremor and its clinical characterization. Thus, one of the hypotheses of this study is that the presence of tremor in the phonatory apparatus during the /s/ production task can differentiate the essential (present tremor) from the dystonic voice tremor (absent tremor) syndromes, assuming that this is a non-phonatory task (production without vocal fold vibration) and that the dystonic voice tremor is task-dependent with tremor manifestation only during speech. It would differ from the essential voice tremor, which exhibits the tremor in any action with posture maintenance.

The objective of this study is to develop a protocol of specific tasks for the evaluation of vocal tremor by nasofibrolaryngoscopy and to identify the tasks that phenomenologically differentiate the syndromes of essential and dystonic tremor.

## Method

### Case series

After approval by the Institutional Ethics Committee under protocol number 1853/10 and the free and informed written consent, this cross-sectional study included subjects with voice tremor treated at the neurolaringology sector of a university hospital. Patient assessment included clinical history, general physical and head and neck examination, auditory-perceptual assessment of voice and nasofibrolaryngoscopy evaluation with specific task protocol.

*Inclusion criteria*: (a) patients aged 18 and older; (b) trembling voice in the auditory-perceptual assessment; (c) tremor of the phonation device structures identified at nasofibrolaryngoscopy; (d) voice tremor complaint for over a year; (e) absence of periods of complete remission of voice tremor.

*Exclusion criteria*: (a) treatment of the tremor or use of antihypertensive beta-adrenergic blockers in the past six months; (b) laryngeal paralysis or morphological lesions in the phonatory apparatus; (c) signs of Parkinson's or cerebellar disease; (d) thyroid hormone alteration.

### Specific task protocol

The nasofibrolaryngoscopy was performed with the patient in the sitting position with no topical anesthesia. Three anatomic subsites (palate, pharynx and larynx) were observed during the following specific tasks: prolonged production of phonemes (/é/, /i/, /s/, /z/), quiet breathing and continuous whistling. The tasks were evaluated for at least 5 s. Phonemes were produced using pitch and intensity that were comfortable for the patient, with the exception of /i/, which was produced in falsetto. The tests lasted an average of 2 min and were carried out with a KayPENTAX FNL-15RP3 flexible endoscope under continuous halogen lighting, connected to a TOSHIBA IK-CU44A camcorder and recorded in digital video system.

### Visual–perceptual assessment of tremor

The video images of nasofibrolaryngoscopy tests were edited in order to identify each specific task performed and remove the audio track to reduce the observation bias. The visual–perceptual analysis of the videos, omitting patient identification, was performed through the consensus of three otorhinolaryngologist examiners with experience in neurolaringology. The evaluators were instructed to: (1) assess the presence or absence of tremor at each subsite evaluated for each of the proposed tasks of the study; (2) identify whether there was a reduction in the intensity or disappearance of tremor in the /i/ task in falsetto in relation to the /é/ task (for evaluation purposes it will be called tremor reduction in falsetto task). There were no limits as to the repetition of the videos for the examiner's assessment.

The criteria used to consider the presence of tremor were: (a) tremor on the palate – rhythmic oscillation of the soft palate; (b) tremor in pharynx – rhythmic constriction of the pharyngeal wall; (c) vertical laryngeal tremor – rhythmic oscillation of the larynx in the vertical plane in relation to the surrounding aerodigestive tract; (d) horizontal tremor of the larynx – rhythmic oscillation of the vocal folds, vestibular folds or arytenoids in the horizontal plane.

To identify specific tasks related to tremor syndromes, initially the patients were classified as having essential and dystonic tremor according to the presence of horizontal tremor of the larynx during the /s/ production, and then this task was combined with others (phonatory and non-phonatory), based on the assessments made by consensus among the three examiners.

### Statistical analysis

In the descriptive analysis of the sample characteristics, the mean, minimum value, maximum value and standard deviation were calculated for numeric variables; for the categorical variables, the absolute and relative frequencies were calculated. Fisher's exact test was used to identify associations between categorical variables of the protocol. The *α* significance level of 5% was used for the conclusions obtained through the inferential analyses.

## Results

Table 1 summarizes the main clinical and demographic characteristics of the study sample.

**Table 1:** Descriptive analysis of the clinical and demographic characteristics of individuals with voice tremor in the assessed sample.

*Number of individuals*	19

*Gender, n (%)*	
Male	2 (10.5%)
Female	17 (89.5%)

*Age at evaluation (years)*	
Mean and standard deviation	66.4 ± 13.1
Minimum-maximum	29–88

*Age at tremor onset (years)*	
Mean and standard deviation	59 ± 15.6
Minimum-maximum	22–82

*Duration of tremor (years)*	
Mean and standard deviation	7.4 ± 5.7
Minimum-maximum	1–24

*Type at onset, n (%)*	
Abrupt	1 (5.3%)
Progressive	18 (94.7%)

*Symptoms, n (%)*	
Voice tremor	19 (100%)
Phonatory effort	15 (78.9%)
Decreased voice intensity	18 (94.7%)

*Tremor improvement factor – alcohol, n (%)*	
No	14 (73.7%)
Unknown	5 (26.3%)

*Tremor aggravating factors, n (%)*	
Coffee	0%
Emotional stress	13 (68.4%)
Physical stress	5 (26.3%)

*Sites of tremor, n (%)*	
Isolated voice tremor	10 (52.6%)
Limbs	8 (42.1%)
Head	3 (15.8%)
Face	4 (21.1%)

The 19 individuals included in the study performed the protocol tasks adequately, except for one patient who failed to perform the continuous whistling. Through the information collected from the visual–perceptual assessment of the tremor by consensus among the three examiners, 12 patients were classified as having essential tremor and seven as dystonic tremor, according to the presence or absence of horizontal tremor of the larynx in the /s/ production task (Table 2).

**Table 2:** Description of the syndromic classification of patients according to the presence of horizontal tremor of the larynx at /s/ production.

	Horizontal tremor of the larynx at /s/ production	
	No	Yes
Number of individuals	7	12
Syndrome	Dystonic tremor	Essential tremor

When studying the association between horizontal tremor of the larynx in the /s/ production and non-phonatory tasks (Table 3), a trend was identified that the individual classified as having essential tremor more often exhibited horizontal tremor of the larynx in the quiet breathing task (*p* = 0.057).

**Table 3:** Association of the essential and dystonic tremor syndromes with the presence of tremor per subsites in non-phonatory tasks.

Tremor per subsites in specific tasks		Horizontal tremor of the larynx at /s/ production				*p*
		No (dystonic tremor)		Yes (essential tremor)		
		*n*	%	*n*	%	
*Quiet breathing*						
Palatal tremor	No	7	100.00%	8	66.70%	0.245^a^
	Yes	–	–	4	33.30%	
Pharyngeal tremor	No	6	85.70%	9	75.00%	>0.999^a^
	Yes	1	14.30%	3	25.00%	
Vertical laryngeal tremor	No	7	100.00%	12	100.00%	b
	Yes		–	–	–	
Horizontal laryngeal tremor	No	6	85.70%	4	33.30%	0.057^a^
	Yes	1	14.30%	8	66.70%	

*/s/ production*						
Palatal tremor	No	4	57.10%	4	33.30%	0.377^a^
	Yes	3	42.90%	8	66.70%	
Pharyngeal tremor	No	7	100.00%	10	83.30%	0.509^a^
	Yes	–	–	2	16.70%	
Vertical laryngeal tremor	No	7	100.00%	12	100.00%	b
	Yes	–	–	–	–	

*Continuous whistling*						
Vertical laryngeal tremor	No	7	100.00%	11	100.00%	b
	Yes	–	–	–	–	
Horizontal laryngeal tremor	No	7	100.00%	–	–	<0.001^a^
	Yes	–	–	11	100.00%	

a Descriptive level of Fisher's exact test.

b No statistical test can be applied.

The horizontal tremor of the larynx in the /s/ production was strongly associated with horizontal tremor of the larynx in the continuous whistling task (*p* < 0.001). In the selected sample, the 11 individuals classified as essential tremor (except one who failed to perform the continuous whistling task) had horizontal tremor of the larynx in the continuous whistling task.

Comparing the phonatory tasks and the horizontal tremor of the larynx in the /s/ production (Table 4), we observed that a reduction of tremor in falsetto in the palate (*p* = 0.020), pharynx (*p* = 0.038) and horizontal larynx (*p* = 0.002) was more frequent in patients classified as having dystonic tremor. No other phonatory task could differentiate the essential from the dystonic tremor.

**Table 4:** Association of essential and dystonic tremor syndromes with the presence of tremor per subsites in phonatory tasks.

Tremor per subsites in specific tasks		Horizontal laryngeal tremor at /s/ production				*p*
		No (dystonic tremor)		Yes (essential tremor)		
		*n*	%	*n*	%	
/*é/ production*						
Palatal tremor	No	–	–	–	–	b
	Yes	7	100.00%	12	100.00%	
Pharyngeal tremor	No	1	14.30%	1	8.30%	>0.999^a^
	Yes	6	85.70%	11	91.70%	
Vertical laryngeal tremor	No	5	71.40%	7	58.30%	0.656^a^
	Yes	2	28.60%	5	41.70%	
Horizontal laryngeal tremor	No	–	–	–	–	b
	Yes	7	100.00%	12	100.00%	

/*i/ production in falsetto*						
Palatal tremor	No	–	–	–	–	b
	Yes	7	100.0%	12	100.00%	
Pharyngeal tremor	No	3	42.90%	2	16.70%	0.305^a^
	Yes	4	57.10%	10	83.30%	
Vertical laryngeal tremor	No	7	100.0%	8	66.70%	0.245^a^
	Yes	–	–	4	33.30%	
Horizontal laryngeal tremor	No	2	28.60%	–	–	0.123^a^
	Yes	5	71.40%	12	100.00%	

*Tremor reduction in falsetto*						
Palatal tremor	No	1	14.30%	9	75.00%	0.020^a^
	Yes	6	85.70%	3	25.00%	
Pharyngeal tremor	No	3	42.90%	11	91.70%	0.038^a^
	Yes	4	57.10%	1	8.30%	
Vertical laryngeal tremor	No	5	71.40%	11	91.70%	0.523^a^
	Yes	2	28.60%	1	8.30%	
Horizontal laryngeal tremor	No	1	14.30%	11	91.70%	0.002^a^
	Yes	6	85.70%	1	8.30%	

*/z/ production*						
Palatal tremor	No	2	28.60%	–	–	0.137^a^
	Yes	5	71.40%	11	100.00%	
Pharyngeal tremor	No	4	57.10%	2	16.70%	0.129^a^
	Yes	3	42.90%	10	83.30%	
Vertical laryngeal tremor	No	5	71.40%	9	75.00%	>0.999^a^
	Yes	2	28.60%	3	25.00%	
Horizontal laryngeal tremor	No	1	14.30%	–	–	0.368^a^
	Yes	6	85.70%	12	100.00%	

a Descriptive level of Fisher's exact test.

b No statistical test can be applied.

## Discussion

Despite the advances in neurolaringology, the distinction between the essential and dystonic voice tremor remains challenging, and this directly impacts the choice among the several types of treatment. For this differentiation, several research methods have been used, among them the auditory-perceptual voice assessment, the transnasal fiberoptic laryngoscopy (nasofibrolaryngoscopy), acoustic measurements and larynx electromyography.^10^, ^13^, ^14^ The common aspect of these assessments is that they usually search for particularities in each syndrome during phonatory tasks (sound production with vocal fold vibration), precisely the activity at which the diseases are most similar. Based on this observation, we tried to identify among different phonatory and non-phonatory tasks (non-vocal productions without vocal fold vibration) the ones that can represent the phenomenology of tremor applied to the phonatory apparatus, by definition, the basic concept that determines its syndromic classification.

The main characteristic of dystonic tremor is that it depends on a specific task to manifest itself, which, in the case of voice tremor, is phonation. In these patients, by definition, the tremor would not be present in non-phonatory situations (productions without vibration of vocal folds), even if there is muscle action to maintain the laryngeal posture, as during the /s/ production. However, patients with essential tremor have muscle tremor when it is contracted, even if it is only to maintain the posture. Thus, the tremor in phonatory apparatus would be present during any activity where there is posture maintenance, regardless of phonation and, therefore, during the /s/ production. Thus, the /s/ production task, which represents a non-vocal fricative phoneme without vocal fold vibration, appears to be a watershed between these two syndromes, since during its performance patients with dystonic tremor would not have tremor, while patients with essential tremor would manifest it. Therefore, this task was chosen to assess variations and differentiate patients, classifying them as having essential tremor syndrome or dystonic tremor syndrome, and also to identify associations between the syndromes and the several protocol tasks (Table 2).

There is still no consensus on the muscle activity of the larynx during quiet breathing. According to a neurolaringology committee report of the American Academy of Otolaryngology – Head and Neck Surgery,^10^ during respiration or phonation there is no real rest in the larynx. These activities are best defined as postural maintenance, which explains the presence of oscillation of the laryngeal structures in essential tremor. During quiet breathing, horizontal tremor of the larynx was identified in 66.7% of individuals in the present case series, classified as essential tremor (Table 3). Despite the controversy, based on electromyographic studies in healthy subjects, Hillel (2001)^11^ demonstrated that the intrinsic muscles of the larynx may be at rest during quiet breathing, an observation that may explain the absence of tremor in one third of these study patients classified as essential tremor. Initially, the presence of tremor in quiet breathing is not required to diagnose essential voice tremor, but this is a sign that suggests the diagnosis as described by Koufman and Blalock (2004).^12^ One of the patients classified as dystonic tremor also had horizontal tremor of the larynx during breathing, a finding that is not expected according to the literature, since this is not an activity that triggers dystonic posture.^12^, ^15^ However, during the thyroarytenoid muscle assessment by electromyography of 13 patients with dystonic tremor, Hillel (2001)^11^ also identified an individual who showed rhythmic activity in this muscle during quiet breathing. Additionally, a rare occurrence that represents 1% of the cases of laryngeal dystonia, is adductor respiratory dystonia, described by Blitzer et al. (1998),^16^ in which dystonic postures in the larynx are present during breathing. Therefore, despite being quite unusual, patients with dystonic tremor who have horizontal tremor of the larynx during breathing can eventually be identified. Another explanation for the presence of horizontal tremor of the larynx during breathing in this patient classified as having dystonic tremor, is the possibility of a mistaken observation of tremor in this task, or even a false negative result in the /s/ production test, and therefore, the correct diagnosis in this case would be essential tremor.

The continuous whistling task was chosen for inclusion in the protocol due to its resemblance to the /s/ production, that is, it is a non-phonatory task in which there is evident posture maintenance in the larynx. In this sample, the horizontal larynx tremor during the whistling showed to be strongly associated with horizontal tremor of the larynx during the /s/ production, with both tasks appearing always equal in relation to the presence of tremor (Table 3). This observation confirms that these tasks are actually phenomenologically similar and adds evidence that the presence of tremor in these situations indicates a diagnosis of essential tremor.

In 2006, De Biase et al.^17^ evaluated patients with adductor laryngeal dystonia using transnasal fiberoptic laryngoscopy and none of the subjects had spasms while performing the whistling. By understanding the dystonic tremor as a variant of dystonia, the expectation is that it will not exhibit dystonic posturing (irregular tremor) during the whistling, as it was demonstrated in this study, in which all patients classified as such showed no tremor. It is important to emphasize that the accomplishment of this task should be a continuous whistling, because the intermittent whistle is associated with laryngeal structure movement that interferes with tremor perception and does not represent posture maintenance.

The literature regarding adductor laryngeal dystonia has evaluated the change in the intensity of spasms in accordance with the variation of the pitch; phonation in falsetto reduces the stimulus for the occurrence of spasms.^17^ Barkmeier and Case (2000)^14^ reported that the high pitch has a direct influence on the reduction of the voice tremor magnitude, and this is due to the separation of the vocal folds. Almost all of patients in the present study, both in the /é/ production as in the /i/ production in falsetto, showed horizontal tremor of the larynx with no statistically significant differences between the tasks, demonstrating that, when isolated, they do not differentiate the tremor syndromes. Among patients classified as essential tremor, 91.7% had no change in tremor when assessed in tasks with normal and high pitch. However, cases of dystonic tremor exhibited significant reduction of tremor in the /i/ production in relation to the /e/ production (Table 4). Therefore, as the /é/ production is emitted with the vocal folds in closer proximity than with /i/ production in falsetto, the more abducted are the vocal folds, the smaller the fluctuations in patients with dystonic tremor.

The /z/ production is important to verify that the phonatory tasks are not useful to differentiate the types of tremor, as only one patient had no horizontal tremor of the larynx during this activity (Table 4). Despite the similarity between /z/ and /s/ productions, since they are fricative consonants produced in the same articulation point (tongue and palate), they differ from each other, as the first occurs in adduction with participation of vocal fold vibration (phonatory or sound), whereas the other does not (non-phonatory). This single difference justifies the importance of phonetic composition in the diagnosis of dystonic tremor, as the oscillation occurs in sound tasks and is absent during non-phonatory tasks. Evidence of similar specific task is found in patients with adductor laryngeal dystonia, which manifests symptoms worsening in sentences with sonorous consonants (b, d, g, v, j, z, m, n) and improvement in sentences with non-phonatory consonants (p, t, k, f, s, ch).^18^, ^19^ In cases of essential tremor, the oscillation is independent from the task (sonorous or silent).^20^

Unlike dystonia, essential voice tremor usually presents as a more widespread disease manifestation, in which the tremor is not restricted to the intrinsic muscles of the larynx and may involve the palate, pharynx, tongue and articulatory muscles.^10^ However, in this sample, both the palate and the pharynx demonstrated the presence of tremor in several tasks, with no significant difference between the groups, demonstrating that the simple presence of tremor in extralaryngeal subsites does not differentiate syndromes. Even the laryngeal vertical oscillation, suggesting tremor of extrinsic laryngeal muscles,^21^ which was expected to be present especially in cases of essential tremor, was not specific either (Table 3, Table 4). This fact could represent a segmental, rather than focal involvement in patients with dystonic voice tremor. However, in the subsites of the palate and pharynx, the only association found was tremor reduction in falsetto in patients with acute dystonic tremor, similar to what occurred with the horizontal tremor of the larynx. Therefore, it is possible that the tremor observed in the palate and pharynx of these patients is not a primary dystonic alteration of this musculature, but secondary to a laryngeal subsystem dysfunction, as the tremor modulation in all structures was directly influenced by the transition between normal and acute pitch, an eminently laryngeal function. This observation indicates the possibility of an interaction between the central nervous system as the tremor generator and the peripheral neuromuscular system, since a probable modulation in the afferent system ends up modifying the tremor in both the larynx and in other structures, such as the palate and pharynx. These hypotheses are related specifically to dystonic tremor, as oscillation modulation was not identified in essential tremor.

The absence of a gold-standard assessment for vocal tremor analysis makes it difficult to assess the reliability of this proposed protocol. As previously discussed, there is a possibility of diagnostic error in this assessment, for instance, the presence of false negative or false positive results in the examination, and therefore one should avoid establishing a diagnosis based on the tremor representation in a single task. To obtain greater reliability when defining the nosological entity in question, the latter should be determined only when the tremor manifestation in different tasks phenomenologically corresponds to the same tremor syndrome. In cases where the tremor manifestation in the several tasks does not point to the same syndrome, the implementation of this protocol becomes less reliable for diagnostic definition.

Although it seems arbitrary to syndromically classify voice tremor by the presence of horizontal tremor of the larynx in the /s/ production, it is justified by the phenomenological basis demonstrated in this study and the associations found in the several tasks, which partly replicate the characteristics traditionally described for each disease. Therefore, the distinction between essential and dystonic voice tremor seems to be feasible, provided that adequate evaluation is performed, including an assessment of the phonatory apparatus by transnasal fiberoptic laryngoscopy, especially during non-phonatory tasks. Moreover, based on higher diagnostic accuracy, it is possible that specific treatments targeted for each type of tremor may allow a better therapeutic response.

## Conclusion

The protocol of specific tasks during transnasal fiberoptic laryngoscopy is a viable method to differentiate between essential and dystonic voice tremor syndromes. Tasks with significant importance for this differentiation are the /s/ production, continuous whistling and observation of reduced tremor in falsetto.

## Funding sources

This study was supported by 10.13039/501100002322Coordenação de Aperfeiçoamento de Pessoal de Nível Superior – CAPES.

## Conflicts of interest

The authors declare no conflicts of interest.
